# Perspective: Food Environment, Climate Change, Inflammation, Diet, and Health^☆^

**DOI:** 10.1016/j.advnut.2025.100504

**Published:** 2025-09-04

**Authors:** James R Hébert, Richard Holmberg, Morgan Boncyk, Geoffrey Scott, E Angela Murphy, Lorne J Hofseth

**Affiliations:** 1 Cancer Prevention and Control Program, University of South Carolina, Columbia, SC; 2 Department of Epidemiology and Biostatistics, Arnold School of Public Health, University of South Carolina, Columbia, SC; 3 Department of Exercise and Nutrition Science, The George Washington University, Washington, DC; 4 Department of Health Promotion Education and Behavior, Arnold School of Public Health, University of South Carolina, Columbia, SC; 5 Department of Environmental Health Sciences, Public Health Research Center, Arnold School of Public Health, University of South Carolina, Columbia, SC; 6 Department of Pathology, Microbiology, and Immunology, School of Medicine, University of South Carolina, Columbia, SC; 7 Department of Drug Discovery and Biomedical Sciences, College of Pharmacy, University of South Carolina, Columbia, SC

**Keywords:** diet, food systems, global climate change, inflammation, ecology, environmental health, UN Sustainable Development Goals

## Abstract

Human activities contribute to large shifts in the global climate, with far-reaching impacts on ecosystems, societies, and human health. Modern food systems—designed to produce convenience foods that tend to have high inflammatory potential—exacerbate environmental degradation and shape the interwoven challenges of climate, nutrition, and health. Over the past 3 decades, extreme weather has worsened, and poor diets have led to more inflammation-related health problems—2 parallel trends that are exposing system-wide weaknesses and harming global health. Is there evidence of a connection between environmental degradation and inflammation? The medical and environmental literatures were searched by combining “climate change” OR “environmental factors” OR “food systems” AND “inflammation” AND “diet.” All permutations of these terms were used, and all terms were searched as both text words and MeSH terms. The literature on inflammation and health is vast (∼750,000 articles in the National Library of Medicine [NLM]) as is the literature on diet and health (>1.8 million articles in the NLM). Interest in global climate change is growing (∼39,000 references in the NLM and >650,000 references in the Web of Science Core Collection). Although the literature at the intersections of diet and inflammation with either climate change or, especially, food systems is small, evidence points to a connection between global climate changes and inflammation operating mainly through food systems. Large-scale industrialized agriculture and other environmental changes that are heating the planet produce food commodities that are causally related to inflammatory processes within organisms. The interplay between individuals’ dietary decisions and system-level decisions regarding food production and processing sets the stage for deepening understanding of connections revealed in the literature and developing a multifaceted approach to address these critical problems that encompass individual behavior change and collaborative initiatives across sectors to effect meaningful change.

**Statement of significance**

Although scientific knowledge on *1*) diet, inflammation, and health; *2*) climate change; and *3*) food systems is extensive, there is virtually nothing written regarding how all 3 may be connected. We propose a connection between personal behaviors associated with diet, food systems, climate change, and inflammation as the major substrate for determining health in human populations.

## Introduction

Beginning at the end of the industrial revolution in the first half of the 19th century, the collective activities of human beings (*Homo sapiens*) have substantially altered the earth’s atmosphere, oceans, and land surface [1]. Approximately 2 centuries later, the ∼8 billion humans currently inhabiting our planet face environmental and health challenges distinct from those faced by the ∼117 billion humans preceding us [2]. Unlike previous ecological periods spanning ∼300,000 years since our emergence as a distinct species, ∼2 million years since the appearance of the *Homo* genus, and billions of years preceding that, humans are the primary agents of massive ecological changes characterizing the current age [1].

With their use of high-energy inputs, industrial-scale agriculture, food processing, and distribution have contributed to climate change [3,4] while also displacing traditional food production methods [[5], [6], [7], [8]]. Trends in global warming are expected to result in long-term declines in photosynthesis efficiency [9,10] and increased evaporation and transpiration [11]. For supply to meet demand, industrialized agricultural practices that use heavy machinery, synthetic fertilizers, pesticides, antibiotics, and genetically modified seeds have led to monocultural farming, agrochemical runoff, and increased greenhouse gas (GHG) emissions [[12], [13], [14]]. Over the last 2 centuries, and especially since World War II, production has shifted from small local farms and gardens to industrial-scale farming and manufacturing of more energy-dense, nutrient-sparse foods [15,16].

In recent years, ultra-processed foods (i.e., category 4 according to the NOVA classification (*nova classificação in Portuguese)* [[17], [18], [19]]) have proliferated in the United States and in global food supplies [[20], [21], [22]]. These foods are industrial formulations typically consisting of >5 (and usually more) ingredients and often include foods also used in minimally processed foods (i.e., extracted from whole foods, such as sugar, oils, and fats) with added salt, antioxidants, stabilizers, and preservatives. As opposed to the use of minimally processed, but otherwise healthy foods (i.e., for example in nutrient fortification), ingredients found only in ultra-processed foods include substances not commonly used while preparing foods in home kitchens and additives whose purpose is to imitate sensory qualities of category 1 (i.e., whole) foods, or culinary preparations of these foods, or to disguise undesirable organoleptic qualities of the final product [18,19,22].

Ultra-processed foods’ share of the global food market has increased markedly in recent decades, contributing to the increased inflammatory capacity of modern diets [23]. Simultaneously, metabolic dysregulation-related diseases, such as type 2 diabetes mellitus [24] and metabolic dysfunction-associated steatotic liver disease (MASLD) [[25], [26], [27]], are more common and are being seen at earlier ages. Diet-associated inflammation also contributes to most other health conditions, including chronic diseases, such as cardiovascular diseases [28,29]; cancers [30,31], including early-onset colorectal cancer [32]; infectious diseases [[33], [34], [35], [36], [37]]; and the immune mechanisms that prevent both infections [38,39] and cancers [40,41]. Consistent with this evidence, a recent umbrella review of meta-analyses on this subject showed overwhelming evidence linking exposure to ultra-processed foods to >70% of all health outcomes examined including all-cause mortality, cancer, and mental, respiratory, cardiovascular, gastrointestinal, and metabolic disease outcomes [42].

The global climate crisis has led to a decline in biodiversity, affecting taxa across all levels of biological organization, from commensal and symbiotic microbiota to entire phyla [43,44], while concurrently amplifying environmental stressors with significant implications for human health. At the same time, it is important to understand the role of ultra-processed foods and the food systems on which they depend in limiting biodiversity [45].

As a species, we have evolved in response to ecological and environmental pressures over hundreds of thousands of years in our lineage and hundreds of millions of years prior to this. Therefore, it is important to understand the nature of the association between environmental stimuli related to extreme weather and organismal inflammation.

In this article, we aim to describe the association between organismal inflammation, a focal point in biomedical research, and environmental stressors driving inflammation, a growing concern among ecologists and agricultural and earth scientists. By elucidating the putative connection between these 2 phenomena, we provide novel insights into the complex interplay between human health and environmental degradation in the modern age and provide potential solutions to address the massive changes that are rapidly spreading across the globe.

## Current Status of Knowledge

### Conceptualizing the connection between climate change and inflammation

**FIGURE 1:**
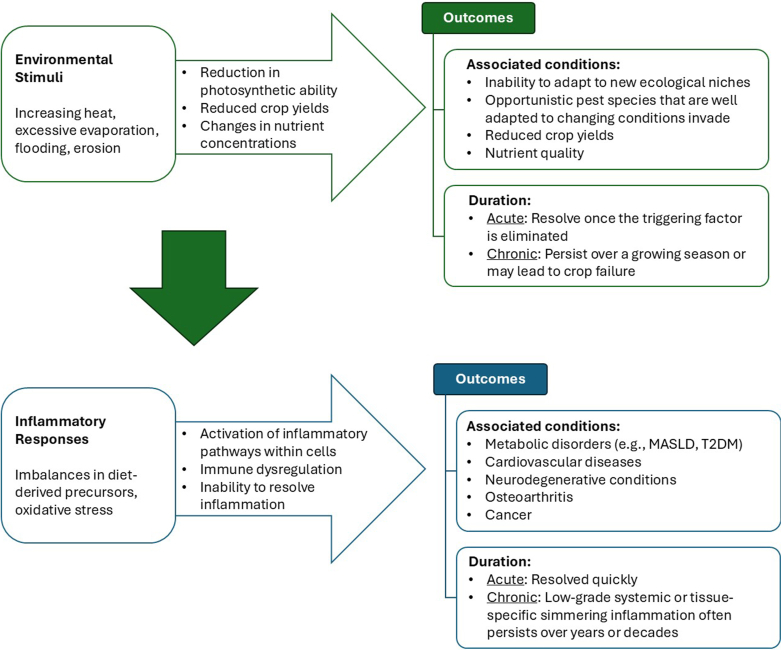
Environmental stimuli and inflammatory responses. MASLD, metabolic dysfunction-associated steatotic liver disease; T2DM, type 2 diabetes mellitus.

Inflammation, characterized by heat, redness, swelling, and pain, is a fundamental physiological response to danger, threat, and injury within organisms [46,47]. Inflammation has been the focus of most biomedical research aimed at understanding how organisms respond to external threats and injury [48]. Environmental triggers associated with overall increases in global temperatures are increasing in frequency and intensity as we observe dramatic increases in GHGs in the atmosphere, rises in ocean temperatures and carbon dioxide concentrations, increased variability in rainfall patterns, and declining plant and animal (ie, both invertebrate and vertebrate species) biodiversity [16,49]. Although these patterns are observed globally, there has been a disproportionate burden placed on the Global South [43,44]. Importantly, although diet modulates inflammation, environmental factors influence conditions for producing food that have important implications for nutrient quality of the diet. All these processes manifest as acute and chronic effects (Figure 1).

**FIGURE 2:**
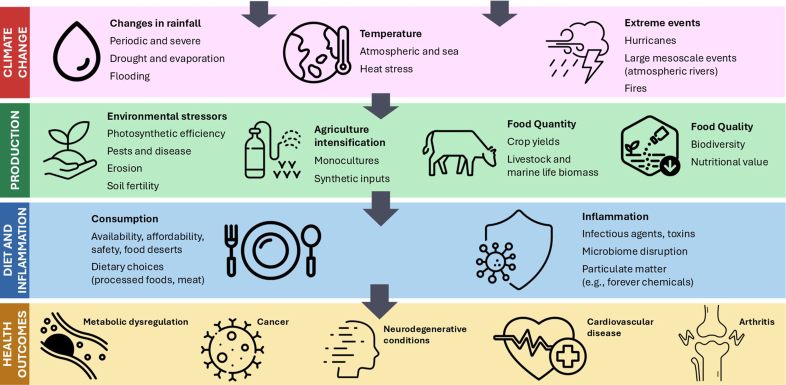
Greenhouse gas-driven climate change, food production, diet, and health. Within each of the 4 domains are listed categories of parameters that constitute major factors of interest that are discussed in the paper. GHG, greenhouse gas.

Environmental changes that disrupt ecosystems and fuel a cycle of events that permeates the natural world are likely to affect human health by promoting inflammatory processes. Although humanity is experiencing chronic systemic and tissue-specific simmering inflammation, partially fueled by diet-related loss of microbiota, environmental effects are being observed at a global scale [50,51]. The world in the modern age is becoming less hospitable to the systems that have sustained life through most of human history [1,3,49]. We make the case that inflammation and environmental changes that have accelerated in the last 75 years are not just correlated; they are causally associated through decisions humans make regarding dietary habits, agricultural practices, and systems of food production (Figure 2).

Human-induced GHG emission continues to rise, along with nonuniform increases in atmospheric and sea temperatures, shifts in rainfall patterns, and a decline in plant and animal biodiversity [43,44,52]. The widespread adoption of radical farming technologies, mainly since World War II and particularly manifested during the Green Revolution, improved food security but has since contributed to environmental degradation [53,54] (Figure 2). Agri-chemical runoff damages environmental systems and negatively impacts human health [[55], [56], [57], [58]]. Synthetic fertilizers and on-farm mechanization are GHG-intensive. Soil carbon levels are reduced when synthetic nitrogen fertilizers are used, hampering carbon sequestration [[59], [60], [61], [62], [63], [64], [65]]. Although the environmental impact of these farming innovations is only now being investigated, it is established that food systems account for one-third of the world’s GHG emissions, and they are proportionally highest in the Global South [4,66].

### Climate, inflammation, and food systems in the modern age

Building upon an understanding of inflammation, as influenced by diet and socioeconomic factors gleaned over the past 70 years, we now turn our attention to the intersection between climate change, food systems, and inflammation. Recognizing that diet is the most important modulator of inflammation and ensuing diseases and disability [67], it is important to consider the broader economic and environmental systems that shape dietary choices. As with most public health threats, there is a disproportionate risk for poor nutritional status among individuals and communities vulnerable to climate disruptions and experiencing food insecurity, which exacerbates environmental, economic, and health-related injustices [68,69]. Individual biological systems (e.g., humans, livestock, laboratory animals) and ecological systems (e.g., whole ecosystems, environments) can be evaluated concurrently to explain the interconnection between climate change, diet, and inflammation across all 5 kingdoms of life (including us and our livestock) (Figure 2). Climatic events affect the production and availability of nutritious food. Climatic events, including drought, floods, hurricanes, rising median and mean atmospheric and sea temperatures, and sea level rise, impact nutrient content of food by influencing plant growth, soil fertility, and nutrient availability [13,[70], [71], [72]]. These changes shape the nutritional landscape and have profound implications for human health and well-being (Table 1) [9,10,[72], [73], [74], [75], [76], [77], [78], [79], [80], [81], [82], [83], [84], [85], [86], [87], [88], [89], [90], [91], [92], [93], [94], [95], [96], [97], [98], [99], [100], [101], [102]].

**TABLE 1:** Specific climate-related effects, mechanisms, and consequences.

Stressor	Mechanisms of impact	Consequences
Drought	Reduced nutrient uptake from soil [[73], [74], [75]]; increased soil salinity [76,77]; wildfires [78]	Nutrient imbalances [72,79], lower overall crop yields [76,77], harmful algal blooms (HABs) and Microcystin [80]
Flood	Mesoscale convection system (MCS), i.e., organized convective storms ≤100 km in diameter [81,82], are an extreme example of massive rapid rainfall that leads to soil erosion [83]; waterlogging and reduced oxygen uptake [84]; agrochemical runoff and contamination [85,86]	Nutrient deficiencies resulting from waterlogging and reduced oxygen [84], pollutants can negatively impact plant health and the nutrient content of the food produced [85,86]
Heat	Temperatures over 30°C impair photosynthesis [9,10], and heat stress increases risk of plant disease, pathogens, and pests [[87], [88], [89], [90], [91], [92], [93]], bacterial growth, and food spoilage [[94], [95], [96]]. High temperatures also deleteriously affect farm worker health and efficiency [98]	Estimated 2% decline in food production per decade until 2050 [68,98], lower crop quality [93,95,[98], [99], [100]], decreased labor productivity [97], less healthy food options, worsening food safety [[94], [95], [96],^98^], increased costs [66,98,101,102]

Climatic events are expected to reduce the nutrient content of food quantity and quality across the supply chain [70,72]. Droughts and floods are expected to affect the availability and absorption of essential nutrients [72,73,83,84]. Droughts may reduce nutrient absorption and cause imbalances, while floods can cause soil erosion, waterlogging, and contamination, resulting in nutrient losses and reduced nutritional value in crops. Exposure to Microcystin, a class of cyanotoxins, poses risks such as liver damage and earlier onset of inflammatory [68] and neurodegenerative diseases [69], with children accounting for 35% of Microcystin exposure illnesses [70]. Extreme temperatures are expected to reduce the availability of safe and affordable nutrient-dense foods by impeding photosynthesis [10,103], inducing heat stress [104], and creating conditions conducive to unwanted bacterial growth [[105], [106], [107]]. Sustainable agricultural practices, such as water conservation, soil management, and crop selection, are essential to mitigate the adverse effects of these environmental events to maintain nutrient-rich food production [74].

### The connection between person-specific inflammation and ecological stress

The biomedical literature on the role of inflammation in health has grown exponentially in the past 3 decades, with peer-reviewed articles doubling every 5 y, totaling over 801,000 in the NLM in mid-July 2025. Diet and inflammation literature has grown even more rapidly. Similarly, global climate literature has grown to over 45,000 references in the NLM and 390,000 in the Web of Science Core Collection. Paralleling academic interest in inflammation and climate change, popular interest in these subjects has grown, with over 6 billion references on inflammation and over 1 billion on climate change resulting from a simple Google search in mid-2025. The proliferation of both the scientific and popular literatures underlines the perceived importance of inflammation as a substrate for numerous mechanisms important to health and the public’s interest in diet and climate change. Although there is interest in the topics individually, there is a dearth of evidence on the interconnection between inflammation in individuals and environmental stimuli driving it.

Before exploring the connection between diet-related decisions, inflammation, and changing ecological systems, it is important to define terms and make important distinctions. First, when referring to food, a recipe, or diet as anti-inflammatory, it is within the context of chronic systemic or tissue-specific inflammation [48]. Although certain foods, such as hot peppers, may initially induce a proinflammatory response, they ultimately (and dependably) reduce long-term chronic systemic and tissue-specific simmering inflammation [[108], [109], [110]]. This may appear counterintuitive, but humans (and other organisms) require an appropriately aggressive inflammatory and immune response to deal with the constant barrage of physical and biological threats, including the trillions of mutations that occur daily [111]. Proinflammatory responses, which occur virtually immediately following an insult, are characteristics of competent organisms that can respond rapidly to danger and usually resolve the initial response within 120 h [112]. Persistent proinflammatory signals, resulting from a failure of ω-3 fatty acid-derived resolvins, protectins, and maresins to resolve the acute response [112], lead to long-term, low-grade chronic inflammation. Second, anti-inflammatory foods that enable an immediate proinflammatory and an appropriately aggressive short-term immune response are naturally colorful, flavorful, nutrient-dense, and calorically sparse [113]. These same foods prevent chronic inflammation over weeks, months, years, or even decades among individuals who consume an anti-inflammatory diet.

Historically, most foods foraged and grown by ancient agriculturalists were anti-inflammatory [109]. Cultivars bred for flavor and/or appearance tend to be more anti-inflammatory than their wild predecessors [114]. Consequently, gardeners or farmers who ate their own produce had anti-inflammatory diets capable of regulating inflammatory responses in a context-dependent manner to ensure appropriate activation or resolution as needed. Even in the modern era, home-grown, whole-food diets are anti-inflammatory. Paradoxically, modern diets tend to be strongly proinflammatory. This shift is largely attributed to promoting calorie-dense, ultra-processed, proinflammatory foods [23,[115], [116], [117], [118]].

Although there are nearly 50,000 articles on diet and inflammation, the literature on environmental factors that influence inflammation is limited, focusing nearly exclusively on the unintentional consumption of dietary contaminants (e.g., pesticides, metals) or the effect of air pollution (e.g., particulates, oxidants) on inflammatory and innate immune responses [119]. As of July 2025, only 5 articles on “environmental inflammation” were included in the NLM database, and they referred either to the tumor microenvironment or the general effect of the environment on the inflammatory response [[120], [121], [122]], an engineering application for implanted devices for cardiovascular repair [123], or improved methods for detecting gastrointestinal cancers [124]. There is virtually no evidence linking personal dietary decisions to large-scale environmental factors. Here, we make the case that environmental factors that result in the breakdown of the natural order/sequence of stimulus and biological response in human physiology have ecological analogs.

From rising temperatures to extreme weather events, climate change disrupts physiological and ecological systems, with cascading effects on human health and well-being. Physiological and ecological systems operate within tolerable ranges, and deviations outside these ranges lead to inevitable losses of efficiency or, at the extreme, breakdown. This decreased efficiency and physiological system breakdown due to environmental stimuli can be observed with hemoglobin and chlorophyll. Hemoglobin carries oxygen most efficiently around 37°C, becoming less efficient as temperatures rise to 41°C, above which people die [125]. Magnesium functions similarly in chlorophyll, with photosynthesis efficiency declining when temperatures exceed 30°C and, especially, above 40°C [9].

Climate change in coastal regions—home to over half of the world’s population—may create unique interactions between microbes and algae. Many coastal ecosystems are eutrophic, with excess nutrients from sources like agricultural runoff [56,96], hypoxia, and rapid microbial and phytoplankton growth. These conditions produce harmful algal bloom (HAB) toxins such as Microcystin [126,127] and highly virulent and antibiotic-resistant *Vibrio* bacteria [106]. In humanized mice, exposure to Microcystin and a high-fat, proinflammatory diet has altered the gut microbiome toward antibiotic-resistant strains that lead to inflammatory responses, contributing to MASLD and affecting the tight junction proteins of the blood-brain barrier [126]. With a third of the United States population obese and at risk for MASLD, additional heat stress associated with climate change may exacerbate these interactions. MASLD and heat stress reduce gut microbiome diversity, alter the relative abundance of gut microbes, and lead to gut leaching by altering tight junction proteins, which leads to concomitant endotoxemia [128]. The gut microbiome strongly influences inflammation and the gut-brain axis [[129], [130], [131], [132], [133]], with important implications for mental health. Although research has focused largely on the gut microbiome/microbiota, soil microbiota may also influence individuals’ gut microbiome [134,135]. These complex interactions underscore the importance of transdisciplinary research. Scientists must work across fields to understand how climate change alters ecosystems—terrestrial, aquatic/marine, and atmospheric—and how this, in turn, affects food systems, diet, and human vulnerability.

As an essential backdrop to global climate change, atmospheric carbon dioxide concentrations have risen from 280 ppm before the industrial revolution to 315 ppm at the start of the Green Revolution to 422.8 ppm in 2024 [71,136,137] and 430.4 ppm in mid-2025 [138]. Anthropogenic carbon dioxide emissions directly degrade nutrient profiles of important C3 food crops, such as maize, wheat, and soy. As atmospheric carbon dioxide levels rise, C3 plants increase carbohydrate content and decrease concentrations of micronutrients and proteins. This increase in carbon dioxide levels may affect the quality of seafood by altering ω-3 fatty acid levels in algae and the organisms that consume them. ω-3 fatty acids are important in regulating inflammatory responses [112] and controlling metabolic diseases such as heart disease [139]. The increased release of carbon dioxide directly correlates with the diminished nutrient density of agricultural products [10,71,75,78], ultimately contributing to higher rates of chronic systemic and tissue-simmering inflammation in humans [140].

### Ecological consequences of industrial agriculture

Many whole foods, such as meats, are now produced in high-input, industrialized settings like feedlots, where animals are raised in crowded conditions and fed concentrated diets to promote rapid weight gain. These foods are calorically dense and low in many anti-inflammatory micronutrients [5,141], particularly anti-inflammatory polyphenolic compounds [[142], [143], [144], [145], [146]]. Industrialized agriculture, particularly meat production, is naturally resource-intensive, relying heavily on water, synthetic fertilizers, and antibiotics, which contribute to water pollution, soil degradation, and GHG emissions [147]. The shift from low-impact grazing to intensive farming exacerbates ecological imbalances with deforestation, habitat loss, and biodiversity decline [148].

To meet rising demand for meat, producers use more feed and hormones, which increases crowding and the use of antibiotics to reduce disease transmission [[149], [150], [151]]. Replacing composting with commercial fertilizers disrupts nutrient cycling, increases erosion, and depletes the soil of micronutrients [72,152], which may ultimately lead to foods that drive unwanted inflammatory responses. At large-scale, soil compaction has dire implications for the soil microbiome [153].

Increased discharge of nutrients and carbon dioxide alters Earth’s major biogeochemical cycles: nitrogen, phosphorus, and carbon. Urbanization exacerbates these effects by disrupting hydrological cycles [154]. Consequently, major ocean regions become hypoxic and eutrophic, leading to dead zones and ocean acidification that cause dead coral reefs [155], thus resulting in reductions in production of ocean fish [156], which tend to be anti-inflammatory [157]. These agricultural practices also increase the risk of drought, erosion, and flooding.

### Industrial agriculture and ultra-processed foods

With some exceptions, ultra-processed foods—as distinct from minimally processed, fortified foods—are generally both energy-dense and nutrient-poor, which contribute to chronic inflammation and poor health outcomes [115,117,158]. These ultra-processed foods have been aggressively marketed to vulnerable populations (e.g., children, low-income individuals), appealing to consumers’ desires through specific organoleptic properties of these food (e.g., sweet taste, bright colors) and reward centers in the brain to increase sales [131,159]. The dominance of foods prepared and consumed away from home, with 73% of the United States food supply ultra-processed [21], reflects the many-decades-long trend toward convenience and mass production [[160], [161], [162], [163], [164], [165]]. Although widely available and affordable, ultra-processed foods have a significant environmental footprint, posing substantial implications for ecosystem health and sustainability [12,16,141,166,167]. This includes concern about microplastics from packaging and processing that are now ubiquitous in environments around the world [[168], [169], [170], [171]], are closely associated with ultra-processing of foods [172], and pose additional health risks (i.e., beyond nutrient inadequacy of the food itself) [[173], [174], [175], [176], [177]].

### The effect of systems-level factors on individuals’ decision-making

The consequences of personal dietary choices are intimately linked to broader environmental challenges, including climate change, habitat destruction, and ecosystem degradation. The trend over the past 3 decades toward examining the effect of dietary patterns, rather than specific nutrients, on various health outcomes, aligns both with an ecologically oriented perspective and with the growing emphasis on nutrient density and nutrient quality rather than solely on calories and quantity [178]. The Dietary Inflammatory Index (DII), widely used (i.e., in over 1900 peer-reviewed articles, including 111 meta-analyses, as of July 2025) to evaluate the effect of diet-induced inflammation on health outcomes, serves as a reliable proxy for nutrient density [[179], [180], [181], [182], [183], [184]]. Addressing these interconnected issues requires a multifaceted approach that promotes sustainable food systems, supports regenerative agricultural practices, and fosters greater awareness of the environmental impact of dietary choices. This will encompass health and nutrition education, access to healthy anti-inflammatory foods that tend to be flavorful, and providing other resources (including culinary literacy). By recognizing the ecological consequences of individual dietary decisions and advocating for more sustainable food consumption patterns, we can contribute to positive environmental change and promote the health and well-being of present and future generations.

The intricate relationship between individual behavior, environmental influences, and broader systemic factors shapes our understanding of health, well-being, and sustainability. Diet-associated inflammation affects cognitive ability [[185], [186], [187]] and psychological health, including depression [[188], [189], [190]], which has become rampant in many societies around the world. Increased prevalence of mental health disorders [[191], [192], [193]] and food allergies [194] may indicate fundamental problems with diet-associated inflammation. The increasing prevalence of these physical and emotional markers may indicate a failing food system. Although available evidence provides a strong rationale for recommending an anti-inflammatory diet, we know that dietary and other health behaviors are informed, shaped, and constrained by a variety of organizational factors. These include aspects over which individuals have some control (e.g., family, faith-community, neighborhood) and others over which they have little or no control. The latter would encompass, for example, availability of healthy food outlets and costs of food as well as housing and public transport, which may have an indirect effect by influencing exposure to environmental effects such as exhaust from internal combustion engines that increase oxidative potential [195] and consequently compromise inflammatory and immune responses [196] by creating an additional demand for dietary antioxidants [197]. Understanding the interplay between personal decision-making and systems-level factors is key to effecting change. The bilateral connection between these factors implies feedback loops. Creating conditions through which individuals can change their dietary behavior may require changes in agricultural and food policy that make change more feasible for a variety of reasons related to convenience, cost, and palatability [3,66,198].

Global climate change has the potential to transform agriculture production, food security, and human health [3]. Climatic events, including increased temperatures, droughts and floods, and rising GHG emissions and carbon dioxide levels, have reduced agricultural production in rural areas [3,199].

Global climate change exacerbates traditional agriculture issues and introduces new challenges in resource allocation, particularly regarding water and fossil fuels, challenges that are compounded by declining photosynthetic efficiency [9,14,43,49,53,75,[200], [201], [202], [203], [204]]. Ocean ecosystems are of major concern due to acidification, HABs, highly infectious microbes, and implications for marine plants [10,[205], [206], [207]], essential for absorbing most of the global excess carbon dioxide from the atmosphere. Additional challenges arise from civil unrest with competition for limited resources. Decisions to resort to war [208] and violence [209] to resolve disputes hinder food production and limit market availability [208].

## Conclusions/Solutions

The current ecological and human health crises require innovative approaches and concerted efforts to effect meaningful change. To address these challenges, we propose a multifaceted approach encompassing individual behavioral changes, policy interventions, and collaborative initiatives across various sectors. These recommendations are consistent with the work of EAT-Lancet Commission indicating that solving both health and food systems problems simultaneously is consistent with literature on both human diet and climate and would represent the most efficient use of resources to improve human health [3].

### Promoting anti-inflammatory diets

Reacquainting people with the organoleptic properties of anti-inflammatory foods can help to effectively drive dietary change. Instead of portion control and dietary deprivation approaches typically (and unsuccessfully) used for weight reduction, innovative approaches, such as acquainting people with the taste, flavor, and texture of anti-inflammatory foods could lead to more effective means of changing diets [[210], [211], [212], [213]]. This approach can build on decades of research into conditioned responses, which nearly universally rely on the unique flavor and taste profiles of specific foods [131,214]. Spices and herbs are the mainstay of these approaches [215,216]. In our own teaching kitchen, we focus on using unique flavors characteristics of anti-inflammatory foods to enhance and expand the experience of eating to encourage sustainable dietary change [217]. In addition to utilizing the organoleptic properties of food, these programs often involve family members and other social contacts (e.g., in communities of faith) to help sustain change and expand program effectiveness [218]. It is conceivable that such programs can be supported through policy changes that would include, among other things, developments in the agricultural and social services sectors.

### Changing incentive structures

Calorie-dense foods are expensive per nutrient content [[219], [220], [221], [222]] and in terms of the environmental degradation entailed in their production [12,167]. However, they are *seen* as inexpensive foods because of subsidies and their widespread availability. In contrast, plant-based diets are nearly always the healthiest and least environmentally damaging [3]. Individuals do not make decisions in isolation. Modifying economic incentives and subsidy programs can encourage consumers to adopt dietary patterns that are more sustainable for human and environmental health. Health insurance providers may find it beneficial to cover healthful diets as they lower health care costs [[223], [224], [225]]. Redirecting subsidies toward nutrient-dense, plant-based foods can make healthy options more accessible and affordable for all individuals [167,225]. Subsidies should account for future climate models, not historical trends. This allows farmers to proactively diversify their crops to meet the needs of a changing climate while also promoting human health. On the demand side, there are some encouraging developments. These include methods to use food prescriptions to support food as medicine programs [198,226,227]. These could be based in primary care settings [228]. One example is a program conducted by our group to provide a “farmacy” program at a federally qualified health center in Orangeburg, South Carolina [229]. Although focusing on individuals with metabolic disease, this also supported local farmers (see next section) by providing an outlet for their produce. Expanding and sustaining such efforts could attract the attention of payers (e.g., government players, insurance companies, and workplaces that self-insure) that may be open to persuasion regarding the evidence demonstrating efficacy and effectiveness of these programs to successfully influence important health outcomes [230]. With a strong empirical foundation in the basic science of inflammation, diet, and health, the field is ripe for conducting innovative studies such as adaptive and pragmatic community and clinical trials [231] that can ultimately pave the way for dissemination and implementation research [232,233]. With changes in incentives, food manufacturers may find lucrative markets for healthier, less intensively processed foods.

### Supporting local agriculture

Investing in local agriculture initiatives, such as community-supported agriculture programs and farmers’ markets, can bolster food security and promote sustainable farming practices. Local production simplifies the food supply chain, reducing food processing and, by association, agricultural waste. Supporting local farmers and producers can help to improve the community’s health [229] by increasing access to fresh, nutritious foods while reducing reliance on environmentally damaging food production methods. However, in planning such programs, thought must be given to the types of foods that come from different sources. For example, locally grown foods will typically be nutrient-dense and, therefore, naturally anti-inflammatory. These foods also tend to be perishable. Supply chain considerations, especially in relation to seasonality, must be considered. Whole-grain commodities will typically come from noncommunity sources. So, sourcing the C3 crops needs to be done with care to avoid the inappropriate use of ultra-processed foods, which are both less susceptible to spoilage and are not typically combined with foods grown in local agriculture.

### Encouraging home gardening

Promoting home gardening initiatives enables individuals to take control of their food supply and cultivate nutrient-rich, anti-inflammatory foods. We have observed some of these effects in our own work [234,235]. As shown in Thailand, Bangladesh, and Zimbabwe, programs designed to encourage home gardening increased the availability of foods rich in beta carotene [236]. Home gardening provides access to fresh produce and promotes physical activity, environmental stewardship, and connection with nature [235,237]. As with other ideas presented, practical realities regarding incentive structures and strategic use of supports need to be addressed in developing, executing, and sustaining these efforts [234,235,238].

### Applying ecological principles

The Gaia Hypothesis provides a rational, holistic approach to solving human health crises associated with inflammation while also addressing agricultural issues ranging from the effect of global climate change on photosynthetic efficiency to promoting soil conservation and organic composting for increasing crop yields and concentrations of anti-inflammatory compounds in foods [239]. These solutions, which address the harmful effects of ultra-processed foods [240] while also providing a framework that takes into account dietary goals and drivers of global climate change [241], have important implications for achieving the United Nation’s Sustainable Development Goals [242].

In conclusion, recognizing the intricate interplay between individual behavior, environmental influences, and broader systemic factors is essential for promoting health, well-being, and sustainability. Diet plays a substantial role in linking environmental changes to inflammatory-driven chronic diseases. By advocating for sustainable food systems and raising awareness of the environmental impact of dietary choices, we can drive positive changes and enhance human health and well-being.

## Author contributions

The authors’ responsibilities were as follows – JRH, RH, MB, GS, EAM, LJH: contributed to and wrote the manuscript; and all authors: read and approved the final manuscript.

## Funding

Drs. Hébert, Murphy, and Hofseth were supported by the United States National Cancer Institute-funded REMEDY Study grant U01 CA272977-01, which is part of the MeDOC Consortium.

## Conflict of interest

The authors report no conflicts of interest.
